# 4D Super-Resolution Microscopy with Conventional Fluorophores and Single Wavelength Excitation in Optically Thick Cells and Tissues

**DOI:** 10.1371/journal.pone.0020645

**Published:** 2011-05-31

**Authors:** David Baddeley, David Crossman, Sabrina Rossberger, Juliette E. Cheyne, Johanna M. Montgomery, Isuru D. Jayasinghe, Christoph Cremer, Mark B. Cannell, Christian Soeller

**Affiliations:** 1 Department of Physiology, Faculty of Medicine and Health Sciences, University of Auckland, Auckland, New Zealand; 2 Kirchhoff-Institute for Physics, University of Heidelberg, Heidelberg, Germany; National Institute of Health, United States of America

## Abstract

**Background:**

Optical super-resolution imaging of fluorescently stained biological samples is rapidly becoming an important tool to investigate protein distribution at the molecular scale. It is therefore important to develop practical super-resolution methods that allow capturing the full three-dimensional nature of biological systems and also can visualize multiple protein species in the same sample.

**Methodology/Principal Findings:**

We show that the use of a combination of conventional near-infrared dyes, such as Alexa 647, Alexa 680 and Alexa 750, all excited with a 671 nm diode laser, enables 3D multi-colour super-resolution imaging of complex biological samples. Optically thick samples, including human tissue sections, cardiac rat myocytes and densely grown neuronal cultures were imaged with lateral resolutions of ∼15 nm (std. dev.) while reducing marker cross-talk to <1%. Using astigmatism an axial resolution of ∼65 nm (std. dev.) was routinely achieved. The number of marker species that can be distinguished depends on the mean photon number of single molecule events. With the typical photon yields from Alexa 680 of ∼2000 up to 5 markers may in principle be resolved with <2% crosstalk.

**Conclusions/Significance:**

Our approach is based entirely on the use of conventional, commercially available markers and requires only a single laser. It provides a very straightforward way to investigate biological samples at the nanometre scale and should help establish practical 4D super-resolution microscopy as a routine research tool in many laboratories.

## Introduction

Single molecule localisation microscopy (known as PALM, STORM, fPALM, etc.) allows far field optical imaging with a resolution of ∼20 nm [1], [2], [3], or ∼1/25^th^ of the wavelength of light. This remarkable increase in resolution was obtained using special switchable dyes and multiple laser excitation sources to limit the number of fluorescent molecules active at any one time to enable the position of each molecule to be determined. Recent advances, however, have enabled localisation microscopy using conventional dyes and labelling methods [4], [5], [6], [7], [8]. The original work was generally limited to single proteins or markers because of problems in availability and/or selectivity in activation of photo-activatable probes. However, the molecular proximity and degree of co-localisation between different protein species are highly important because of their criticality in molecular signalling. Therefore extending localisation microscopy to multiple labels greatly enhances the utility of this high-resolution method [9], [10], [11].

A variety of approaches may be used to distinguish markers, including sequential imaging of two (or more) marker species [10], [12], [13] and use of specific excitation wavelengths to selectively excite individual markers [10]. By spectrally resolving the signal from a single fluorescent molecule from a mixed population into two (or more) channels and detecting both simultaneously, problems arising from illumination and time dependent changes in the specimen and location drift are minimised [9], [11]. In addition, the cross talk (or ambiguity in molecule identity) is reduced because the emitted spectrum can only arise from a single molecule at one time (which is, of course, also the key to the improved spatial resolution in the method).

The three-dimensional (3D) nature of most biological samples has been minimised by employing either total-internal reflection fluorescence (TIRF) excitation [14] or thin sectioning [14] in localization microscopy. This helped to avoid autofluorescence and also ensured that the fluorescent emission from the molecules of interest were sharply focussed. The Airy disk was then interpreted solely in terms of its centroid, giving a position in the x-y plane and all 3D information was lost. More recently, the problem of 3D localization has been has been addressed by optically engineering the point spread function to encode 3D information [15] or recording from 2 focal planes simultaneously [16]. However, as one uses thicker specimens, the problem of out-of-focus fluorescence as well as autofluorescence reduce contrast and make localisation more difficult.

In this study we have developed a novel 4D imaging approach we call “d^4^STORM” (in analogy to the original dSTORM technique [7]) that is able to provide information on the spatial location of individual marker molecules in multiply labelled biological specimens. Because the method uses readily available and inexpensive parts, this advance should lead to increased utility of localization microscopy for co-localization of biologically important molecules. Our approach differs from previous work by combining 3D and spectral super-resolution simultaneously with readily available fluorochromes as well as operating in a wavelength range where biological autofluorescence is minimised.

## Results

**Figure 1:**
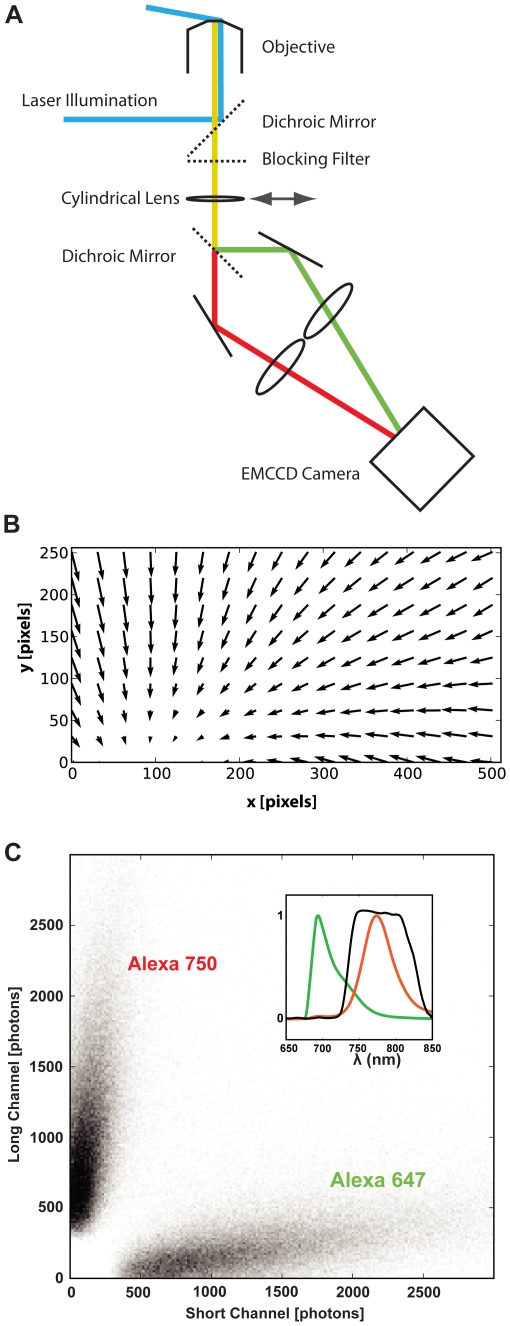
Schematic depicting optical setup for ratiometric localisation imaging. **A.** A single laser at 671 nm is used to provide excitation and the collected light is split into two bands using a dichroic mirror. The bands are imaged side by side on an electron multiplying CCD (EMCCD). An optional cylindrical lens allows astigmatism based 3D localisation. **B.** A vector field that shows the distribution of lateral chromatic shifts between the channels measured with a bead calibration sample. This vector field was used for chromatic shift compensation during fitting of the single molecule events as detailed in the Methods. The longest arrows shown correspond to a shift magnitude of ∼90 nm. **C.** Single molecule events are observed as flashes with an intensity component in each channel. When these intensities are plotted against each other, discreet populations emerge corresponding to each fluorochrome in the sample. One such plot, obtained from a sample in which neurons had been transfected with GFP-alpha-sap97 and subsequently labeled with antibodies against GFP (Alexa 647 secondary) and synapsin (Alexa 750 secondary) is shown. Inset: Recorded emission spectra of Alexa 647 (green) and Alexa 750 (red), the black trace is the transmission curve of the dichroic mirror in the splitter device.

A single inexpensive 671 nm diode laser could induce reversible photochemical conversion [7], [17] in a number of near-infrared fluorescent dyes including Alexa 647, 680, & 750 that are commercially available as antibody conjugates. The stochastic ‘blinking’ of the fluorescent labels was enhanced by a ‘switching’ buffer containing a primary thiol [7] (for details see Materials and Methods). Single molecule events were detected in two channels by placing a dichroic mirror into the detection path. This split the image into two components which we call here ‘short’ (680–740 nm) and ‘long’ (740–8301 nm) channels. The images at these wavelength were digitized with a single electron multiplying charge-coupled device (emCCD) camera by focussing each of the two images simultaneously onto two halves of the camera (see Figure 1A). To add axial position encoding, a cylindrical lens was used to introduce astigmatism in the images [15].

Figure 1B shows measurements of the spatial non-uniformity in the displacement between the two separate images. It was notable that although generally small (<90 nm), the displacement field was highly non-planar. This prevented simple alignment of the localization events in both channels by a single correction factor, and probably arose from residual aberrations present in the dichroic mirror and focusing lenses. Nevertheless, having measured the displacement field, we found that it was invariant from day-to-day and could be used to correct the images (to better than 10 nm) to allow spectral classification of fluorescent single molecule blinks and accurate co-localization measurements.

With this system, events from a sample containing both Alexa 647 and Alexa 750 fluorophores were generally detected in both channels. When the relationship between both channels was plotted, two clearly separated populations were visible (Figure 1C). The spectral ‘crosstalk’ in conventional microscopy, as given by the mean ratios, would be approximately 15% in both channels. However, with single molecule detection the signal could only have come from one fluorophore at a time, and we can therefore assign the signal arising on both channels to one particular fluorophore with much higher certainty [9]. The high degree of separation between the two populations in Figure 1C immediately suggests that more than two labels can be assigned simultaneously using only two detection channels [11]. In practice, we determine the intensity components of single molecule events in the two detector channels and assign molecular identity on the basis of a probabilistic model. This model takes the uncertainty in the ratio arising from photon collection statistics into account and also uses information on the ratios of the dyes present in the sample (see Materials and Methods and Supplementary Text S1). Tests with actual samples showed that the error in molecular assignment using our probabilistic approach was ∼0.4% (see Materials and Methods).

**Figure 2:**
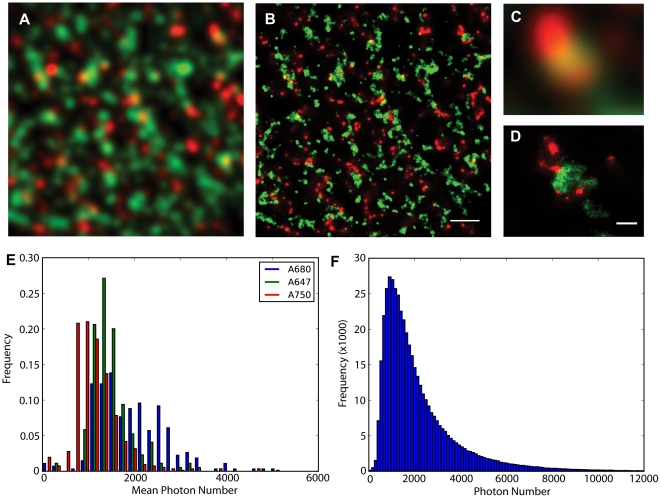
Two-dimensional super-resolution imaging of the distribution of Ryanodine receptors (red) and Caveolin (green), using Alexa 680 and Alexa 750 secondaries, in the periphery of isolated rat cardiac myocytes and overview of dye properties. Panel **A** shows the sample at conventional resolution, panel **B** the super-resolved image. Comparison of enlarged detail (**C** & **D**) shows that apparent overlap in the diffraction-limited images is not seen in the corresponding super-resolution image. **E**. Histogram of mean photon number per event of a dataset of ∼400 ratiometric super-resolution images. The mean photon numbers were calculated for each image in the dataset, the histogram of actual photon numbers per single molecule event are shown in panel **F**. Scale bars B: 1 µm, D: 200 nm.

This method of almost cross-talk free dual-colour super-resolution imaging was used to investigate the spatial relationship between two types of protein, the membrane protein caveolin-3 (CAV3) and the ryranodine receptor (RyR), a protein located on the sarcoplasmic reticulum, near the surface membrane of cardiac ventricular myocytes [18]. Using indirect immunofluorescence labelling and the dye pair Alexa 680 and Alexa 750 to label RyR and CAV3, respectively, we obtained dual colour super-resolution images (Figure 2A,B). Both proteins form clusters or aggregates and many areas that appear to contain co-localizing structures at conventional resolution (Figure 2A) often showed little overlap in the corresponding region of the super-resolution image. The increased information in the super-resolution data was used to calculate improved estimates of co-localizing protein fractions. Based on the diffraction limited data (Figure 2A) 28.6% of CAV3 were co-localized with RyRs while the co-localizing fraction in the super-resolution data (Figure 2B) was only 4.9%, indicating that the apparent partial co-localization between the proteins at diffraction-limited resolution is almost entirely due to optical blurring.

The accuracy of these super-resolution methods is heavily dependent on photon numbers, so we also analyzed the photon yields per single molecule event for the three dyes (Alexa 647/Alexa 680/Alexa 750) used for our super-resolution imaging. Figure 2E shows the result of analyzing a database of 400 super-resolution images (containing a total of >46×10^6^ single molecule events). Typically, mean photon numbers ranged from ∼1000 to ∼2000 per blink using frame integration times of ∼50 ms, compatible with typical lateral localisation accuracies of ∼15 nm (std. dev.), see also Supplementary Figure S2. With a typical average photon yield of 2000 photons/event (representative of results obtained with Alexa-680, see Figure 2F), it should be possible to simultaneously detect up to 5 different labels with <2% crosstalk (see Supplementary Figure S3).

**Figure 3:**
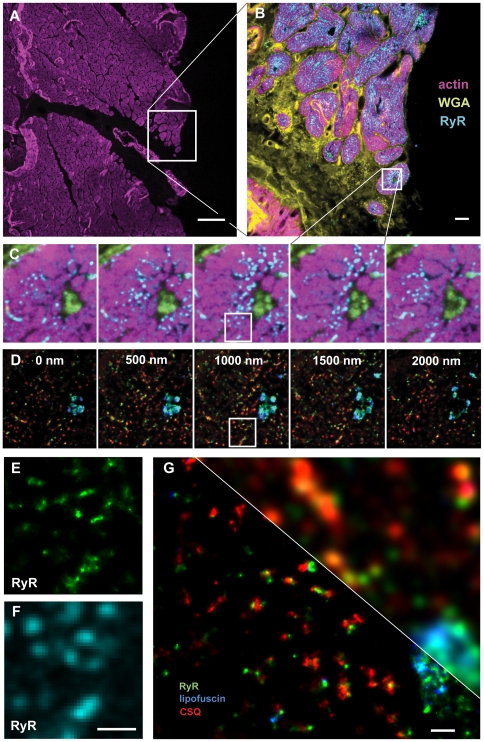
Correlative confocal and super-resolution imaging of a human cardiac tissue section. The section was ∼10 µm thick and was labeled with phalloidin for f-Actin (Alexa 488), WGA for the cell membrane and extracellular matrix (Alexa 594), along with antibodies for the ryanodine receptor (RyR, Alexa 680) and calsequestrin (CSQ, Alexa 750). In addition to the applied labelling, a strong endogenous fluorescence signal from lipofuscin was recorded. The shorter wavelength labels (Actin, WGA, and RyR) were imaged on a confocal microscope, and the sample then taken to the localisation microscope where super-resolution imaging of the longer wavelength labels (RyR, lipofuscin, CSQ) was performed. Panel **A** shows an overview of the cellular structure across a large tissue area that is indicated by the actin labeling (largely muscle cell contractile protein). Scale bar 100 µm. Panel **B** is a projection of a confocal stack taken of the region indicated in A. Scale bar 10 µm. Panel **C** shows a confocal stack of a small detail area from B and panel **D** shows an optically sectioned super-resolution stack, within the region covered by the confocal stack in C. Panels **E** & **F** compare corresponding confocal (F) and super-resolution (E) images both using the RyR-Alexa 647 signal. Note the good correlation between the data and the improvement in resolution in E. Scale bar 1 µm. **G**: 3-colour super-resolution image of a small area in the tissue sample, note the improved resolution as compared to the conventional resolution image section. Since the ratios of Alexa 647 and lipofuscein were relatively close some crosstalk did occur. Scale bar 500 nm.

The ability to use localisation microscopy in fixed tissue and spectrally distinguish several markers was investigated using thick (∼10 µm) tissue sections from human heart. The sections were labelled with Alexa 488 phalloidin (for actin filaments) and Alexa 594 wheat germ agglutinin (WGA) (for extracellular matrix and cell membranes). In addition, we labelled intracellular proteins with indirect immunofluorescence with Alexa 647 and Alexa 750 for cardiac ryanodine receptor (RyRs) and calsequestrin (CSQ) respectively. With confocal microscopy, low magnification overview images of large tissue areas (Figure 3A) as well as smaller regions at diffraction-limited resolution (Figure 3B,C) were used to select several cells for multicolor super-resolution imaging. After transfer of the sample to a super-resolution microscope the signal from the two near infrared stains (labelling RyR and CSQ) and an intrinsic signal from lipofuscin (pigment granules that are present in the aging heart [19] which was also simultaneously excited at 671 nm) generated sufficient contrast for good single molecule detection of the three markers (Figure 3D–G). Lipofuscin blinking was supported by the dSTORM mountant, since samples mounted in pure glycerol exhibited an ∼10-fold reduced lipofuscin event rate during illumination at 671 nm. These data also illustrate the benefit of using near IR dyes as fixed tissue autofluorescence was reduced by almost two orders of magnitude in the regime >680 nm as compared to the visible range at ∼500 nm (see Supplementary Figure S4, Supplementary Text S2).

In most previous work, tissue imaging by localisation microscopy relied on ultra-thin cryosections [3] but we were able to acquire z-sectioned lateral super-resolution stacks in these 5–10 µm thick sections (with diffraction-limited axial resolution, see also Figure 3D). Overall, 5 markers were imaged in these tissue samples, 3 in ratiometric super-resolution mode and 2 markers were acquired at diffraction limited resolution. The RyR (Alexa 647) signal was imaged using both conventional confocal microscopy (excited at 633 nm) and super-resolution and showed good correlation between both imaging modalities (Figure 3E,F).

**Figure 4:**
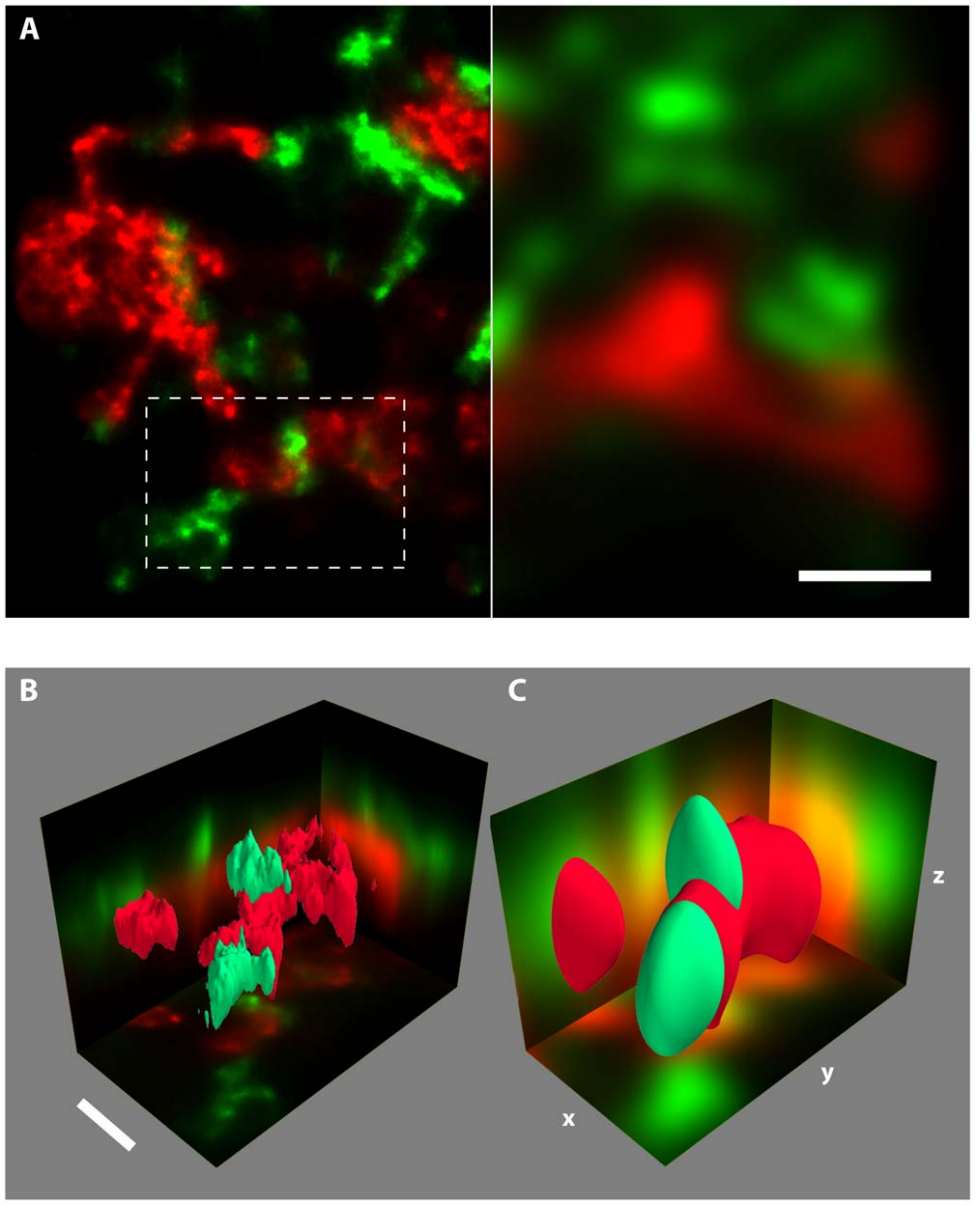
3D super-resolution imaging of GFP-alpha-SAP97 (antibody labelled with an Alexa 647 secondary, green) and Synapsin (with an Alexa 750 secondary, red) in a primary hippocampal culture. 4D imaging was performed using dual-colour 3D localisation based on astigmatism in conjunction with the ratiometric multi-colour approach. **A**. Comparison between detail in a super-resolution image (left) with the corresponding conventional diffraction limited image (right) of the two proteins. **B** & **C**. 3D rendering of the region indicated in A using super-resolution (B) and conventional resolution (C). Note that in the high resolution data (B) ‘lateral’ as well as ‘axial’ synapses can be distinguished. The 2D images shown on the axes are average projections along the respective directions. Scale bars A: 1 µm, B: 500 nm.

Ratiometric multi-colour localisation microscopy can be extended to full 3D localisation by introducing a cylindrical lens, analogous to single colour imaging [15]. With this setup (see also Figure 1A) we imaged neuronal cells in a primary hippocampal culture in which the pre-synaptic protein synapsin and the postsynaptic protein SAP97 were labelled with Alexa 750 and Alexa 647 (respectively). Figure 4 shows dual-colour 3D localisation images where synapses between axially adjacent processes are clearly resolved in the 3D localisation data, while difficult to discern at conventional resolution (Figure 4H). In tissue samples, an axial resolution of better than 65 nm (std. dev.) could be routinely achieved with a lateral resolution of ∼15 nm (std. dev.), corresponding to a PSF volume of <0.1 aL, a volume ∼300 times smaller than in diffraction-limited optical microscopy.

## Discussion

Using standard immuno-fluorescence protocols, commercially available secondary antibodies, and a relatively simple optical setup involving only a single excitation laser that simultaneously excites several infra-red fluorochromes, we have been able to obtain 4D spatial-spectral super-resolution images. The 4^th^ dimension is provided by what we call “spectral super-resolution” because the ratiometric approach with single molecule data allows us to fit more distinguishable markers into a given spectral region than would be possible with conventional diffraction-limited imaging. The simplicity of both the equipment and the conventional sample preparation mean that this super-resolution approach, based on photochemical conversion of the fluorochromes to long-lived dark states, is not more demanding than high quality confocal imaging, and the equipment costs and complexity are probably lower than for a confocal microscope.

### Ratiometric 4D super-resolution imaging with conventional near-infrared dyes and a single excitation laser

A great advantage of the ratiometric method is the virtual elimination of cross-talk between markers with suitably different emission channel ratios, here we identified the pairs Alexa 647/Alexa 750 and Alexa 680/Alexa 750 with cross-talk typically below 1%. This relies on the single-molecule nature of the events which ensures that an observed signal arises exclusively from a fluorophore of one specific colour, unlike the situation in conventional imaging where an unknown quantity of each fluorescent species is present within a diffraction limited volume. By comparison, cross-talk is more difficult to avoid when using sequential activation [10], [12], [13], [20] where cross-activation or thermal relaxation of photoswitched markers may complicate the interpretation of protein-protein proximity in biological samples.

An important practical aspect of using the ratiometric method is the careful characterization and correction of chromatic shifts between detection channels. We observed lateral chromatic shifts on the order of ∼100 nm with our custom-built splitter, similar to that obtained with a commercial splitter-device (Optosplit II, Cairn, UK, data not shown). While field shifts of that amplitude are generally not a problem during diffraction-limited imaging, if left uncorrected in super-resolution work, would broaden detected molecule ratios and interfere with co-localisation and distance measurements at the nanometre scale (as noted elsewhere, e.g. [21], [22]). The chromatic shifts were readily corrected with a software based compensation procedure using a 2D vector field (in the axial direction it was sufficient to apply a constant axial shift across the whole field). As axial and lateral resolution in LM are further improved a full vectorial 3D compensation field can be applied [22].

Previous ratiometric approaches used either special photo-switchable dyes [9] or were limited to visible fluorochromes [11] which, in our hands, has been problematic for single-molecule based super-resolution imaging of tissue sections (see below). Both studies used labelled bead samples to estimate the distribution of expected channel intensities for each fluorescent species. We simplify this method by using intensity uncertainty information returned by the single-molecule fitting process along with an analytical prior (essentially assuming a fixed ratio for each marker species, see Supplementary Text S1) to calculate probability based measures from which to distinguish the marker populations. This has the practical advantage of not requiring a separate calibration measurement, and facilitates adaptation to sample dependent changes in event intensities or spectra. An in-situ calibration can be performed automatically by performing either peak detection or cluster analysis of the observed amplitude ratio distribution to determine the actual mean dye ratios.

The use of a single excitation laser to simultaneously excite all super-resolution labels greatly simplifies the optical setup and also the acquisition protocol which does not need to control activation laser intensity and switching during the frame series. It also eliminates the need for specific cross-talk elimination procedures that can be required in activation-colour based multi-labeling schemes [20].

### Volumetric imaging and 3D localization in ratiometric super-resolution imaging

Full 3D localization is compatible with ratiometric detection by using one of the point-spread-function engineering methods such as astigmatism or helical PSF approaches [15], [23]. Here we used astigmatic detection and could routinely achieve axial resolution of ∼65 nm (std. dev.). An important practical factor that allowed us to obtain high quality single-molecule super-resolution data in extended volumes was an acquisition protocol that moved the focus repeatedly axially through the desired imaging volume during acquisition (advancing axial position every ∼100 frames). This minimized time dependent bleaching effects within the image volume which is essential for any quantitative analysis of label density.

### Super-resolution imaging of tissue sections with conventional dyes

While in previous work tissue imaging by localisation microscopy generally relied on ultra thin cryosections [3], we were able to image single molecules in optically thick (∼5–10 µm) tissue sections. Recently, it has been shown that Alexa 647-activator dye pairs (STORM probes [1]) can be used for STORM super-resolution imaging of tissue sections [20]. Here, we have extended the ability of super-resolution imaging of tissue sections to using conventional fluorescent probes with the straightforward dSTORM approach [7]. We show that, besides Alexa 647, several other commercially available dyes in the red emission region, such as Alexa 680 and Alexa 750, have the required single molecule brightness and photostability For this purpose, an additional advantage of using dyes that emit in the range >650 nm is the greatly reduced tissue autofluorescence which, we suggest, is critical to the usefulness of these dyes for single molecule based imaging of formaldehyde fixed tissue samples.

Using these dye combinations, we have demonstrated straightforward 4D super-resolution imaging in tissue sections using only commercially available probes, an approach we call “d^4^STORM” in analogy to the original dSTORM technique [7]. The relative simplicity of this approach should help increase the widespread use of super-resolution imaging. It should also make many sample types accessible that have not been previously investigated with these high-resolution methods, including clinical samples as demonstrated here with human cardiac tissue samples.

### Correlative confocal and super-resolution imaging

The ability to perform diffraction-limited confocal imaging of several labels simultaneously increased the ability to identify and select areas in which to perform super-resolution imaging. This correlative confocal and super-resolution imaging was performed with Alexa-647 labels. It is notable that the excitation intensities and dwell-times associated with the acquisition of high signal-to-noise ratio confocal data did not induce extensive permanent photobleaching that would have prevented subsequent super-resolution imaging. In the experiments reported here, the samples were transferred between dedicated confocal and super-resolution setups but in principle both modalities could be implemented in the same instrument. This would allow rapid selection of structures in a known tissue context (e.g. scar boundaries or tissue areas exhibiting structural changes in disease) to conduct targeted super-resolution imaging. Additionally, given suitable confocal detectors the correlated confocal imaging could be extended to other near-infra-red super-resolution labels such as Alexa 680 and Alexa 750.

Finally, we note that the methods that we developed here could be modified to be compatible with live cell imaging by, for example, using glutathione as a thiol to induce reversible photochemical conversion and blinking [24]. It is also possible that other infra-red dyes might be used, such as oxazine based fluorophores [24], if the cells depend on oxygen for survival. Even allowing for the relatively low time resolution of full dSTORM imaging, hybrid methods such as uPAINT that can be used to track large numbers of molecules in live cells [25] should benefit from our approaches to enable live cell multi-colour tracking.

## Materials and Methods

### Isolated Rat Ventricular Myocytes

Cardiac myocytes were enzymatically isolated as described [26] in accordance with protocols approved by the University of Auckland Animal Ethics Committee (approval R330). Isolated cells were fixed in PBS containing 2% paraformaldehyde (PFA; w/v) for 10 minutes, washed and labelled according to standard immuno-fluorescence protocols [27], [28] with a rabbit polyclonal anti-CAV3 antibody (Cat no. AB2912; Abcam, MA) and a mouse monoclonal anti-RyR2 antibody (MA3916; ABR, CO). Highly cross-adsorbed Alexa 680-linked goat anti-mouse IgG and Alexa 750-linked Goat anti-rabbit IgG secondary antibodies (Invitrogen, NZ) were applied for 2 hours at room temperature.

### Human Cardiac Tissue Sections

Human cardiac tissue was obtained with the written informed consent of the family of the organ donor as approved by the New Zealand Health and Disability Ethics Committee (approval NTY/05/08/050). Samples were taken from the midline region of the left ventricle, fixed with 1% paraformaldehyde in PBS overnight at 4°C, cryoprotected in 30% sucrose, frozen in liquid nitrogen chilled isopentane and stored at −80°C until further processing. 5–10 µm thick frozen sections were cut on a Leica CM 1900 cryostat and mounted on coverslips for subsequent antibody labelling.

Sections were hydrated in PBS, blocked for 1 h with FX signal enhancer (Invitrogen), and then incubated with antibodies against the cardiac ryanodine receptor (RYR2) (MA3-916, Thermo Fisher Scientific) and calsequestrin (PA1-913, Thermo Fisher Scientific) overnight at 4°C. Sections were washed three times in PBS and incubated in phalloidin Alexa Fluor 488, wheat germ agglutinin Alexa Fluor 594 and secondary antibodies labelled with Alexa Fluor 647 (RyR) and Alexa Fluor 750 (calsequestrin) (Invitrogen) for 1 h at room temperature. Antibody solutions consisted of 1% BSA in PBS. Sections were washed three times in PBS.

### Neuronal Culture

Hippocampal cultures were prepared, transfected and immunostained as described previously [29]. Briefly, hippocampal P0 rat hippocampi were dissociated (as approved by the University of Auckland Animal Ethics Committee) with papain and plated onto poly-D-lysine coated coverslips. Cultures were transfected at 12–13 days in vitro with αSAP97-EGFP [30] via calcium phosphate precipitation. Neurons were fixed with 3.7% formaldehyde 24–48 hours after transfection and immunocytochemistry to detect synapsin-I (BD Pharmingen 611392, mouse, 1∶500) and GFP (Abcam AB13970, chicken,1∶1000) was performed. The secondary antibodies used were goat anti-mouse IgG Alexa Fluor 750 (Invitrogen, 1∶300) and goat anti-chicken IgG Alexa Fluor 647 (Invitrogen, 1∶300).

### Mounting and embedding for localisation imaging

Stained tissue sections, neuronal cultures and isolated myocytes were mounted by adding 20 µl of a “switching buffer”[7] (0.5 mg/mL glucose oxidase, 40 mg/mL catalase, 10% wt/vol glucose, 50 mM β-mercaptoethylamine, 20% PBS and 80% glycerol, all obtained from Sigma–Aldrich) onto a number 1.5 coverslip carrying the specimen, a slide was then placed on top, and the edges sealed with nail varnish.

### Experimental Setup

Images were acquired on a Nikon TE2000 inverted microscope with a Nikon 60×, 1.49NA oil immersion TIRF objective (Nikon, Japan), and an Andor IXon DV887DCS-BV electron multiplying CCD camera (Andor Technology, UK). Custom illumination optics allowed high intensity laser illumination at an adjustable angle, and a custom objective holder coupled directly to the microscope stage minimised thermal and mechanical drift. Focussing was provided by a piezo focusing unit (Physik Intrumente P-725, Germany). Laser illumination was provided by a single solid state laser (Viasho VA-I-N-671, China) providing a focal plane intensity of ∼10^9^ W/m^2^ at 671 nm over a 10 µm diameter field of view. This intensity is somewhat (∼5–10x) higher than that reported for dSTORM measurements on thin specimens (eg [5]). Part of this increase (a factor of ∼2) compensates for reduced excitation of Alexa 647 & 750 at 671 nm. The remaining effective increase is useful when progressing to tissue imaging as it increases the proportion of out of focus molecules in the dark state at any given time and hence improves contrast. The illuminator was adjusted to generate a highly inclined light sheet [31].

Between the microscope and the camera a splitter device (Figure 1A) was added, allowing us to image two spectral channels side by side on the CCD chip. The dichroic mirror (FF741-Di01, Semrock, Rochester, NY) used to separate the channels was centered at 741 nm.

For 3D localisation, a pair of 1 diopter astigmatic lenses (from an optometrist trial set) were inserted into the optical path after the standard fluorescnce filter changer. By differential rotation of the two lenses, the degree of astigmatism could be adjusted allowing us to balance z-localisation accuracy against in-plane contrast. For the experiments shown in this paper, the axes of the lenses were at an angle of ∼85 degrees to each other, resulting in an effective focal length of the cylindrical component of ∼6 m [32].

### Chromatic Shift Calibration

When performing multicolour localisation microscopy, it is important to account for differential chromatic shifts ≥10 nm. We measured the shift vector field using a field of 200 nm dark-red fluorescent beads. The difference in the apparent positions of each bead in the two colour channels was then measured, resulting in a set of shift vectors each associated with the position of a bead in the image. The sample was moved and the process repeated several times to get good coverage of the entire field of view. To obtain an interpolated shift value for any image location, 2D smoothing splines were fitted to the x- and y-components of the shifts measured at bead locations (see Supplementary Figure S1).

This approach can in principle be extended to include the axial component of the shift, however, the comparatively poorer axial resolution (∼65 nm, std. dev., with 3D localisation) meant that we were able to use a constant value for the axial shift. This value was estimated by imaging a thin layer consisting of a mixture of Alexa 680 and Alexa 750 secondary antibodies dried onto a coverslip, and calculating the difference in the mean axial positions of the single fluorophores.

### PSF Measurement

For 3D localisation, measured PSFs were acquired by aligning and averaging the images of several fluorescent beads in a widefield image stack (axial spacing 50 nm). 200 nm dark red fluorescent beads (Invitrogen) were used. The size of the 200 nm beads means that they provide only an approximate representation of the true PSF. It has been shown that such an oversized PSF estimate results in only a minor degradation in the precision of single molecule localisation [33], an effect which is likely to be offset by the improved signal to noise obtained by using larger beads.

To confirm that the effect of the slightly oversize PSF on localisation ability was small, we also performed experiments in which we deconvolved the PSF estimate with the bead shape in order to obtain a more realistic estimate of the true PSF. To perform this deconvolution we used a Richardson-Lucy [34] solver implemented in python. For our experimental data we could not detect any difference in mean localisation precision when localisation was performed with deconvolved versus non-deconvolved PSFs.

### Image Acquisition

Because a single laser is used for the excitation of all spectral signatures, the image acquisition protocol is considerably simpler than that used in many previous studies. A region of interest was located using arc lamp illumination, the laser turned on and images streamed to disk at a rate of 20 Hz. For our illumination conditions and in a viscous mountant (which slows diffusion and thus chemical interactions) this provided a good match between frame time and single molecule event duration, optimising the event signal to background ratio. At the beginning of each acquisition, 20 dark frames were acquired with the laser turned off. An initial period of bleaching/shelving (typically 1–2 s) was also performed with the camera EM gain set to zero (to avoid saturation of the gain register) after which the EM multiplication was turned up to a value corresponding to a gain of ∼35. Between 20000 and 50000 frames were acquired in each series.

Where appropriate, Z Stacks were obtained by moving the objective using the piezo focuser. In contrast to conventional microscopy where each complete z section is acquired sequentially, we moved the focus repeatedly through the stack over the course of the acquisition. This has the advantage minimised bleaching artefacts within the image stack.

### Event detection

Before performing position determination, single molecule events must be detected in the noisy data frames using a filter tailored to detect point like objects. When detecting events for 2D localisation, images were correlated with a Gaussian, followed by a non-uniform background correction in which a strongly filtered copy of the image is subtracted. For 3D localisation, Wiener filtering with a projection of the measured PSF was performed rather than correlation with a Gaussian. The choice of a Wiener filter was motivated by flexibility – the Wiener filter will also produce a clearly defined central peak for more exotic 3D PSFs such as the double-helix [23] or phase-ramp [35] PSFs whilst being sufficiently computationally inexpensive to be applied to each frame.

The images thus obtained were subjected to an adaptive thresholding algorithm based on a scaled estimate of the SNR in each pixel (obtained by taking the square root of the raw pixel photon numbers). The images were then labelled using a binary labelling routine and the centres of intensity of each region calculated to use as starting positions for a fit. The event detection was performed independently for both halves of the CCD (the two colour channels). Note that this event detection procedure is only used to decide which regions of the image contain active molecules, the subsequent fitting is performed on the raw image data.

### 2D fitting

2D fitting was performed as in [4], but using a modified model function to allow ratiometric measurements and to take chromatic shift into account. A small region of interest (11×11 pixels) was extracted from each of the two colour channels at the location of each detected event. This was then fit using a Levenberg-Marquardt solver and the model function:
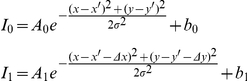
where 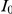
& 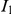
are the raw pixel intensities in the two channels, 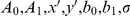 the fit parameters, and 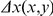 and 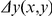 the previously measured chromatic shift for that position.

### 3D fitting

3D fitting was also performed on the raw image data using a Levenberg-Marquardt algorithm. In order to be able to extract 3D position information, we used a model function which generated candidate 2D images at arbitrary 3D shifts by interpolating into the measured PSF using cubic spline interpolation. This is similar to the procedure described in [33], [35] but with the model function adapted for chromatic shifts (similar as described for 2D fitting) to allow both colour channels to be fit simultaneously and the measured chromatic shifts to be included.

### Postprocessing and Data Visualisation

#### Filtering

Events having an intensity, estimated position error, or width outside reasonable bounds were rejected, allowing us to eliminate spuriously detected events and to obtain z-sectioning/out of focus rejection.

#### Separation of colour channels

Each single molecule event was assigned to a particular fluorescent species based on the ratio of its intensity in the two colour channels. However, rather than simply segmenting the ratio space (see description in Results), the probability of the event originating from a particular fluorescent species was calculated from an analytical detection model which took the observed channel intensities and their estimated errors into account (see Supplementary Text S1). This approach provided probabilities for deciding whether the label intensity fit into either channel or was due to some other fluorophore.

Spectral cross-talk was estimated by measuring the fraction of events detected as belonging to the Alexa 750 species when imaging a sample containing only Alexa 680. Approximately one in 250 events were incorrectly identified placing an upper bound on the cross-talk of ∼0.4%.

#### Visualisation

After colour channel assignment, density map images were generated from the point positions for each channel using the jittered triangulation based described elsewhere [36]. 3D images were computed using a generalisation of the triangularisation method using tetrahedra. For 3D sectioned images in which 2D positions are calculated at different defocus (as opposed to full 3D localisation), each point was assigned a z-position corresponding to the focus position at the time it was detected. In computing the triangulations, these z-positions were randomly jittered with a std. deviation equal to the slice thickness (200 nm) to better reflect the true uncertainty in z, and to avoid triangularisation problems arising from large numbers of co-planar points. x & y positions were jittered by neighbour distance as described in [36]. For images obtained using full 3D localisation, the z coordinates were jittered by their estimated localisation accuracy.

For the creation of figures the resulting images were post processed with ImageJ to make contrast and brightness suitable for display. Synthetic diffraction limited images were obtained as follows: Figure 2A – single molecule events were rendered as Gaussians with a width equal to their fitted std. deviation. This is equivalent to adding together all the single molecule images, but produces an image with improved signal to noise, better contrast, and optical sectioning due to the rejection of both noise and out of focus fluorescence. The rejection of out of focus fluorescence occurs because single molecule events that are out of focus will be either too faint to be detected, or will be rejected on the basis of their lateral size in the post-processing steps. Figure 4A – as 3D fitting does not return a width, the approach used in 2A could not be applied. The distorted nature of the PSF used to allow axial localisation also means that summing images or rendering events as a copy of their fit will give a poor comparison image. Instead, a sum projection of the rendered high-resolution image was taken (all events were collected from a single focus position) and convolved with a Gaussian kernel with a size equal to that of a widefield PSF.

### Co-localization analysis

The distribution of CAV3 at the surface of single myocytes was characterized in relation to regions of RyR labelling. A binary mask of RyR-labelled regions in 2D dual colour diffraction-limited images was constructed using the adaptive thresholding algorithm described previously [27]. The patches of RyR labelling in localization data were binarized using the cluster segmentation approach described in Baddeley et al. [18], where single molecule events separated by no more than 30 nm were grouped into clusters. Two-dimensional Euclidean distance maps constructed from the RyR masks in either diffraction-limited data or localization images were used for calculating the percentage of CAV3 labelling as a function of the distance to the edge of the nearest RyR cluster.

## Supporting Information

Figure S1 A typical chromatic shift field, showing the measured *x* (**A**) and *y* (**B**) components of the chromatic shift between the two splitter channels as measured using 200 nm far-red fluorescent beads. The magnitude of the shift is given by the colour scale. Panel **C** shows the shifts as a vector field. The shape of this field suggests that there is a difference in the effective magnification as well as a small rotational component between the two channels.
(PDF)

Figure S2 Distribution of localisation accuracies obtained for Alexa 680 linked to secondary antibodies in a typical sample (mean number of photons  = 1800).
(PDF)

Figure S3 The number of spectral channels able to be resolved at a fixed photon number. The width of a point cloud associated with a spectral channel (see Fig 1 in the main article) decreases as the photon count increases, thus allowing more channels to be independently resolved. In order to make a quantitative estimate of our resolving power we measured the width (std. deviation) of this point cloud as a function of photon count for a sample labelled with only Alexa 680. The width of a channel tells us the spacing (in ratio space) that is required to keep crosstalk between channels within a given bound. The number of channels can then be inferred by calculating how many times we can fit this spacing into the interval [0,1), our possible ratio space. We show these curves for 2 choices of separation, representing different values of allowable crosstalk. In calculating the width of the point cloud, bins were chosen to contain a constant number of events (100), resulting in smaller bins (and more noise) towards the low-photon number end of the curves.
(PDF)

Figure S4 Autofluorescence excitation spectrum of PFA fixed cardiac myocytes. Please note that the vertical scale is logarithmic. The straight line shown is an exponential fit to the autofluorescence signal portion that is above the device noise floor and should capture the “trend” of reducing autofluorescence with increasing excitation wavelength.
(PDF)

Text S1 Assigning events to colour channels.
(PDF)

Text S2 Measurement of tissue autofluorescence.
(PDF)
