# Supplementary figures and images for: 4D Super-Resolution Microscopy with Conventional Fluorophores and Single Wavelength Excitation in Optically Thick Cells and Tissues
Source: PLoS One. 2011 May 31;6(5):e20645. doi: 10.1371/journal.pone.0020645 (PMC3105105; doi:10.1371/journal.pone.0020645)

**A**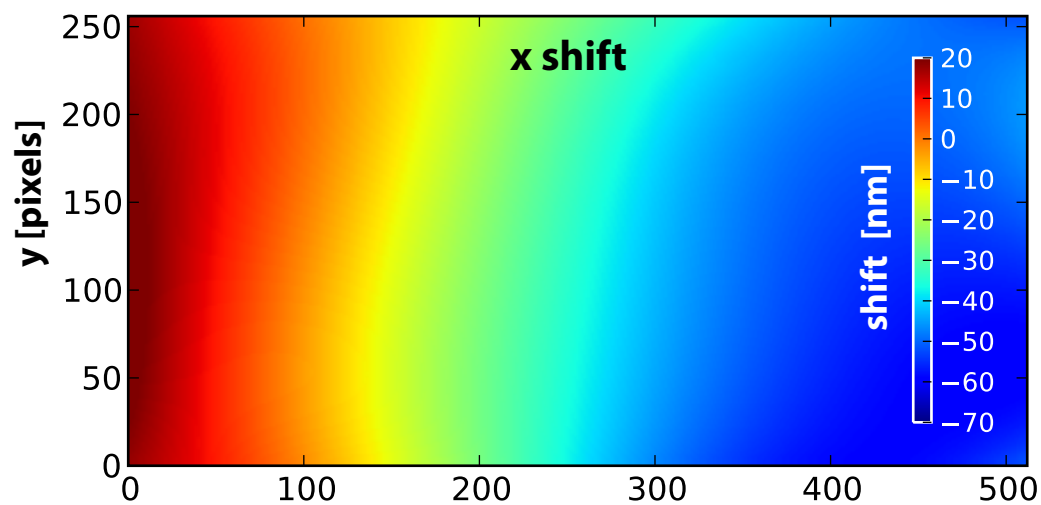**B**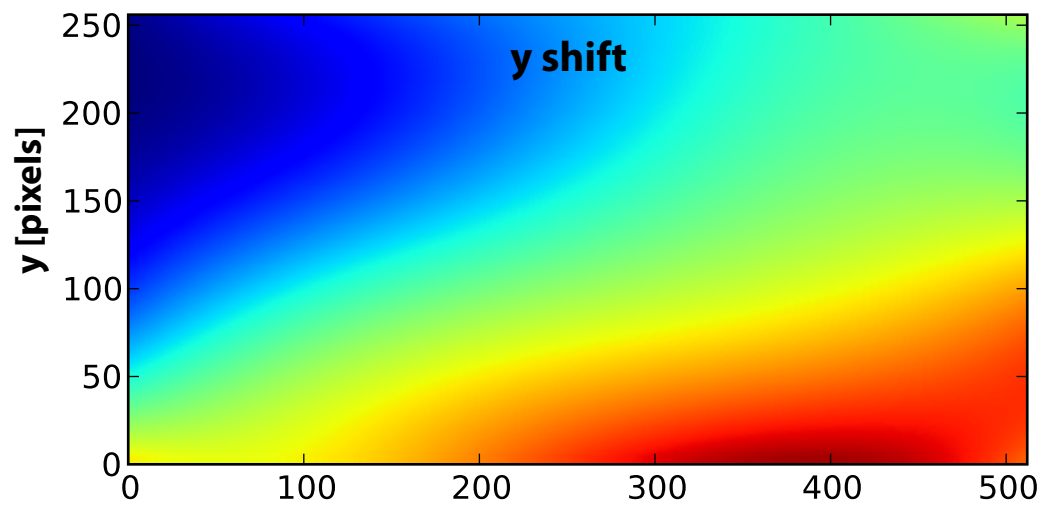**C**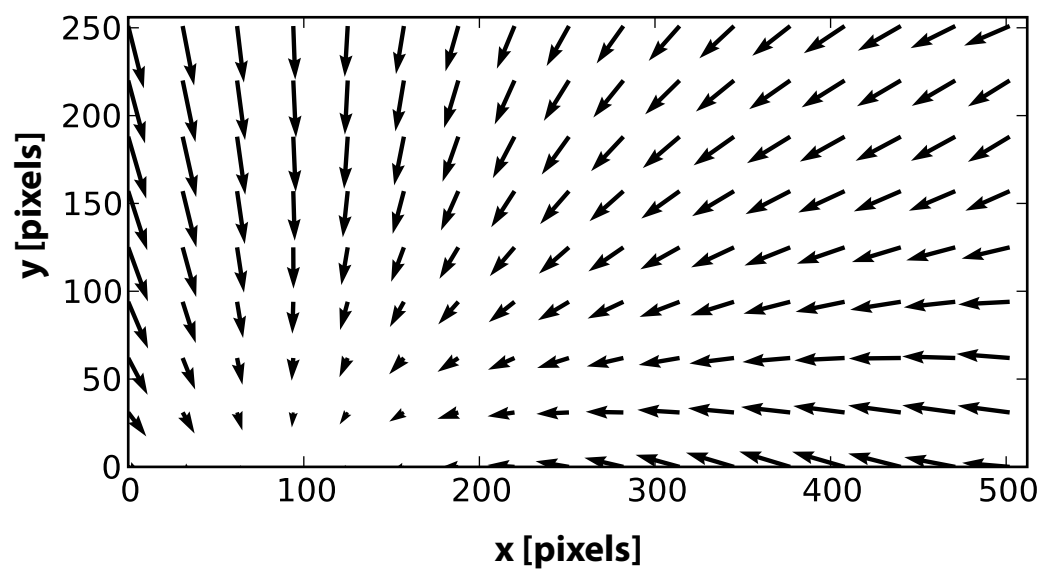

Supplement: Figure S1 — A typical chromatic shift field, showing the measured x (A) and y (B) components of the chromatic shift between the two splitter channels as measured using 200 nm far-red fluorescent beads. The magnitude of the shift is given by the colour scale. Panel C shows the shifts as a vector field. The shape of this field suggests that there is a difference in the effective magnification as well as a small rotational component between the two channels. (PDF) [file pone.0020645.s001.pdf]

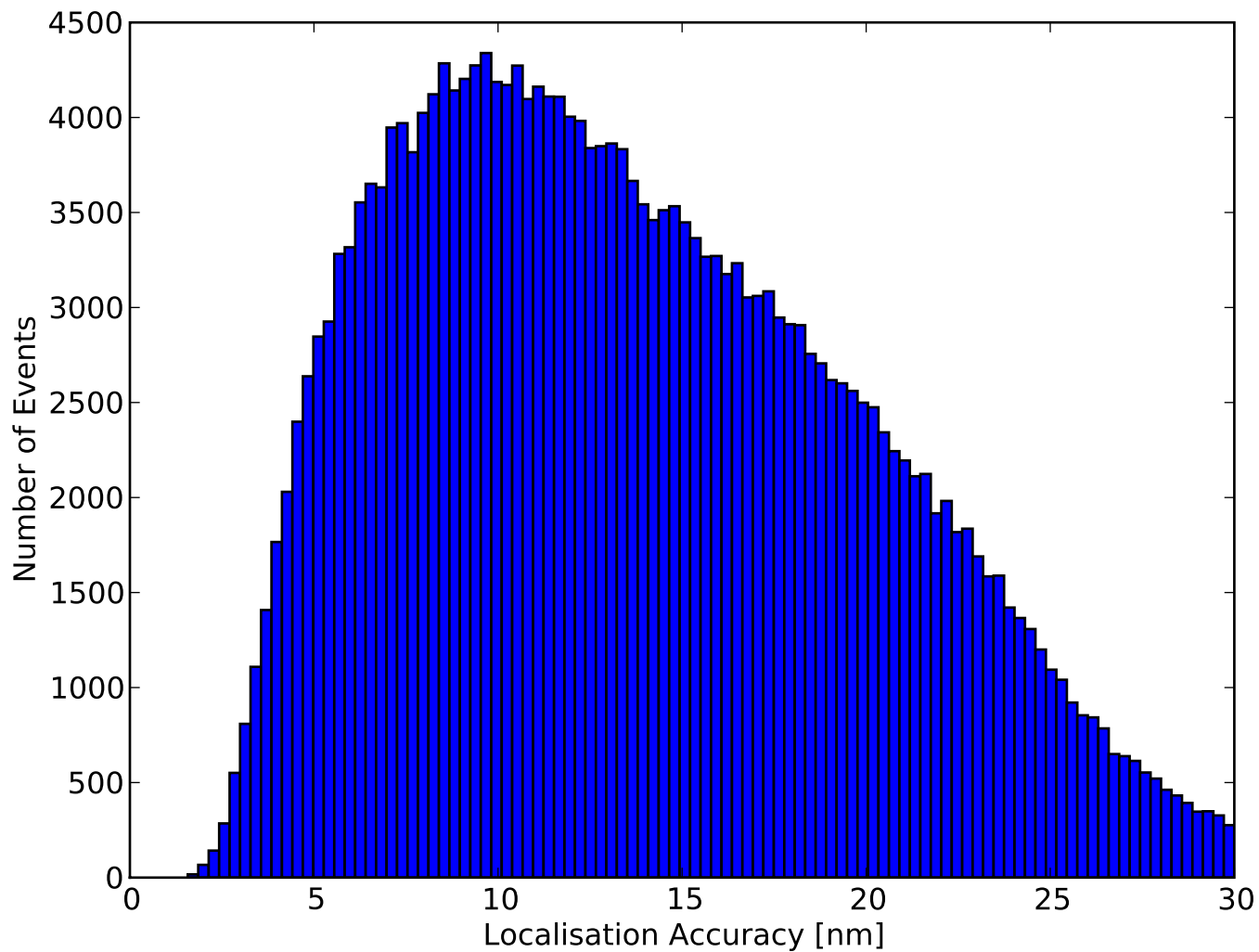

Supplement: Figure S2 — Distribution of localisation accuracies obtained for Alexa 680 linked to secondary antibodies in a typical sample (mean number of photons = 1800). (PDF) [file pone.0020645.s002.pdf]

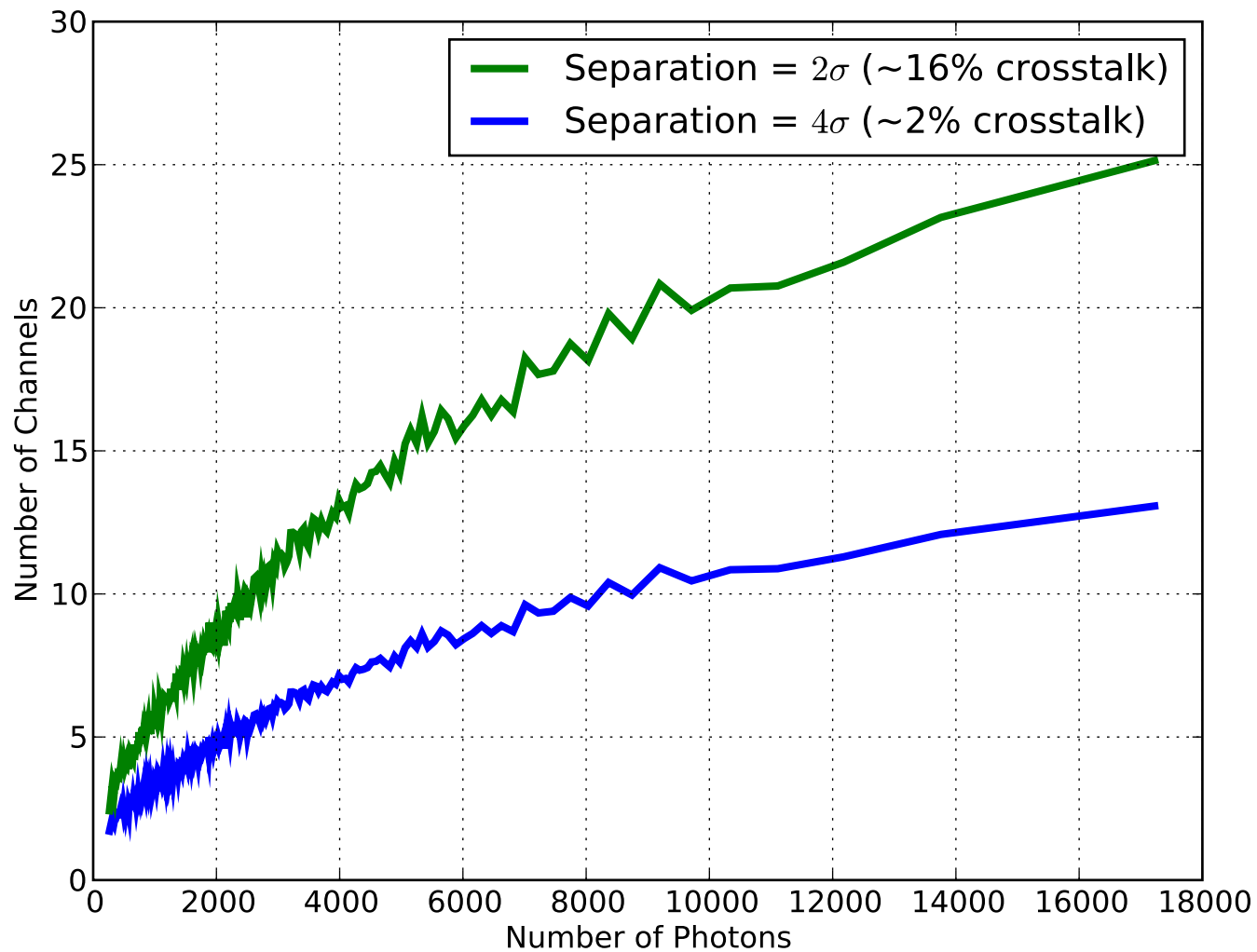

Supplement: Figure S3 — The number of spectral channels able to be resolved at a fixed photon number. The width of a point cloud associated with a spectral channel (see Fig 1 in the main article) decreases as the photon count increases, thus allowing more channels to be independently resolved. In order to make a quantitative estimate of our resolving power we measured the width (std. deviation) of this point cloud as a function of photon count for a sample labelled with only Alexa 680. The width of a channel tells us the spacing (in ratio space) that is required to keep crosstalk between channels within a given bound. The number of channels can then be inferred by calculating how many times we can fit this spacing into the interval [0,1), our possible ratio space. We show these curves for 2 choices of separation, representing different values of allowable crosstalk. In calculating the width of the point cloud, bins were chosen to contain a constant number of events (100), resulting in smaller bins (and more noise) towards the low-photon number end of the curves. (PDF) [file pone.0020645.s003.pdf]

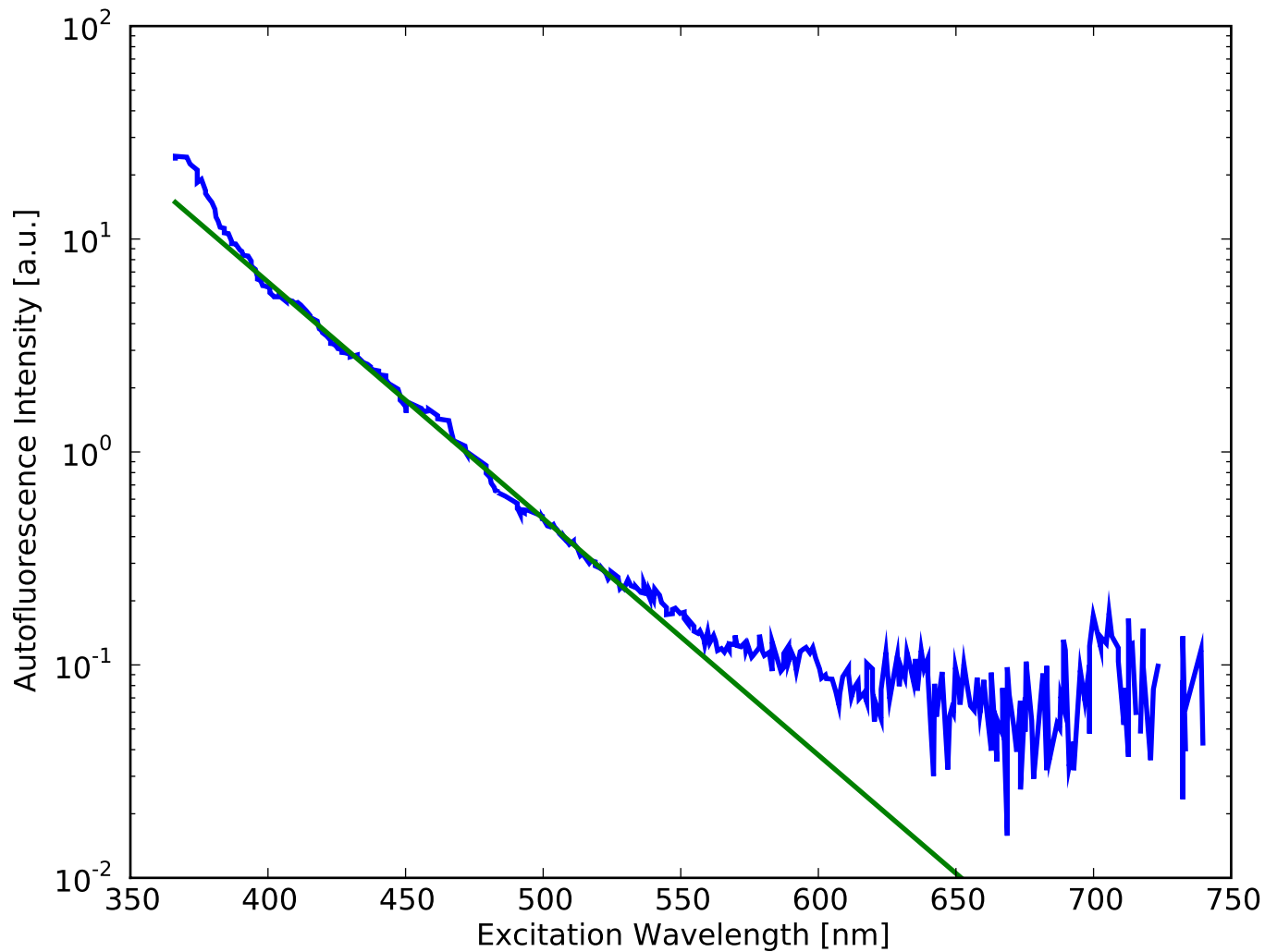

Supplement: Figure S4 — Autofluorescence excitation spectrum of PFA fixed cardiac myocytes. Please note that the vertical scale is logarithmic. The straight line shown is an exponential fit to the autofluorescence signal portion that is above the device noise floor and should capture the “trend” of reducing autofluorescence with increasing excitation wavelength. (PDF) [file pone.0020645.s004.pdf]
