# Supplementary material for: 4D Super-Resolution Microscopy with Conventional Fluorophores and Single Wavelength Excitation in Optically Thick Cells and Tissues
Source: PLoS One. 2011 May 31;6(5):e20645. doi: 10.1371/journal.pone.0020645 (PMC3105105; doi:10.1371/journal.pone.0020645)
Supplement: Text S1 — Assigning events to colour channels. (PDF) [file pone.0020645.s005.pdf]

Assigning events to colour channels

Our fitting procedure provides us both with a position for each single molecule event and with that event's intensity (A_{short} and A_{long}) in each of the two channels. From this pair of intensity values we need to decide which dye the event was most likely to originate from. Each of these values has an associated error, due mainly to the Poisson nature of the imaging process (which predicts an intensity error equal to the square root of the number of detected photons) but also in part to additional factors such as pixelation and inaccuracies in the fitted model. Although the shot noise contribution is expected to follow a Poisson distribution, the intensities are such that this can be well approximated with a Normal distribution with a suitably chosen mean and std. deviation. Our fit algorithm provides error estimates (σ_{short} and σ_{long}) which are obtained from shape of the goal function at convergence and give a good indication of the actual (not purely photon-limited) measurement uncertainty. If single molecule positions were, however, to be estimated using alternate methods (eg centroid estimation) which do not give error estimates, the approximations $\sigma_{\text{short}} \approx \sqrt{A_{\text{short}}}$ and $\sigma_{\text{long}} \approx \sqrt{A_{\text{long}}}$ should be reasonable.

Starting with this information, namely A_{short}, A_{long}, σ_{short} and σ_{long}, we can proceed as follows:

Given a true event intensity A and dye splitting ratio $r = \mathbb{E}[A_{\text{short}}/A]$, and assuming a normal distribution for the intensity error, we can express the probability of having observed A_{short} and A_{long} as:

$$p(A_{\text{short}}|A, r, \sigma_{\text{short}}) = \frac{1}{\sqrt{2\pi\sigma^2_{\text{short}}}} e^{-\frac{(rA - A_{\text{short}})^2}{2\sigma^2_{\text{short}}}}$$

$$p(A_{\text{long}}|A, r, \sigma_{\text{short}}) = \frac{1}{\sqrt{2\pi\sigma^2_{\text{long}}}} e^{-\frac{\left((1-r)A - A_{\text{long}}\right)^2}{2\sigma^2_{\text{long}}}}$$

The joint probability of having observed both A_{short} and A_{long} for a given true total intensity A and splitting ratio r thus follows the relationship:

$$p(A_{\text{short}}, A_{\text{long}}|A, r, \sigma_{\text{short}}, \sigma_{\text{long}}) \propto e^{-\frac{(A_{\text{short}} - rA)^2}{2\sigma^2_{\text{short}}} - \frac{(A_{\text{long}} - (1-r)A)^2}{2\sigma^2_{\text{long}}}}$$

where the normalisation factors have been omitted.

Approximating A with $\hat{A} = A_{\text{short}} + A_{\text{long}}$, assuming a uniform prior on $r \in [0, 1]$, and normalising, one arrives at the following expression for the probability that a given event had a true ratio r:

$$p_r = p(r|A_{\text{short}}, A_{\text{long}}, \sigma_{\text{short}}, \sigma_{\text{long}}) = \kappa e^{-\frac{(A_{\text{short}} - r\hat{A})^2}{2\sigma^2_{\text{short}}} - \frac{(A_{\text{long}} - (1-r)\hat{A})^2}{2\sigma^2_{\text{long}}}}$$

where the normalisation factor κ is given by:

$$\kappa = \frac{\sqrt{2}\left(A_{\text{short}} + A_{\text{long}}\right)\sqrt{\sigma_{\text{short}}^2 + \sigma_{\text{long}}^2}}{\sqrt{\pi}\sigma_{\text{short}}\sigma_{\text{long}}\left[\text{erf}\left(\frac{A_{\text{short}}\sqrt{\sigma_{\text{short}}^2+\sigma_{\text{long}}^2}}{\sqrt{2}\sigma_{\text{short}}\sigma_{\text{long}}}\right) + \text{erf}\left(\frac{A_{\text{long}}\sqrt{\sigma_{\text{short}}^2+\sigma_{\text{long}}^2}}{\sqrt{2}\sigma_{\text{short}}\sigma_{\text{long}}}\right)\right]}$$

We now consider the case that a number N of spectrally distinct dyes are known to be present in the sample, indexed as dye_i with r_i being the splitting ratio of dye_i, and the index i ranging from $1, .., N$. Because of the uncertainty (photon noise) in our measurements, a given A_{short} and A_{long} pair could reasonably have been observed for a range of different underlying splitting ratios. The probability of any given ratio, r, will thus be small and we cannot simply plug the expected ratio for a given dye (r_i) into the formula to obtain the probability of the event having originated from that dye. Instead we need to introduce additional information in the form of a prior describing which ratios are possible. We base this on the fluorescent species we know to be present, and it has the form:

$$p(r|\text{labelling}) \propto \sum_{j=1}^{N} \delta(r - r_j) + c$$

where c represents a (small) constant background term. Including this information and normalising brings us to the following expression for the probability p_{dye_i} that an event was originating from a molecule of dye_i:

$$p_{\text{dye}_i} = \frac{p_r(r_i)}{\sum_{j=1}^{N} p_r(r_j) + c}$$

with p_r defined as above.

For a given dye species, it is also useful to define p_{other_i}, the probability the event originated from the most likely of the other dyes present:

$$p_{\text{other}_i} = \max\, p_{\text{dye}_j}\ j \in [1, N], j \neq i$$

To make the final decision as to which dye species an event originated from, we set bounds on the permissible values of both p_{dye_i} and p_{other_i}, the probability the event originated from the most likely of the other dyes. Various factors such as spectral differences in dye molecules and subtle focal shifts between the two channels may result in a slight broadening of the theoretical dye ratio and thus could lead to false rejections if the bound on p_{dye_i} is too strict. An approach which sets a relatively generous lower limit on p_{dye_i}, but relatively strict bounds on p_{other} allows channels to be 'greedy' and grab the maximum number of events when well spaced, but still offers good rejection when channel spacing is closer (i.e. when the ratios r_j of some dyes are relatively close). We typically use $p_{\text{dye}} > 0.1$ and $p_{\text{other}} < 0.1$.

The principal difference between this approach and simply choosing the most likely dye species ($p_{\text{dye}_i} > p_{\text{other}}$) is that we reject events that cannot reliably be assigned to any dye

species. Such events are typically found at the low end of the event intensity spectrum, and whilst only a small fraction of the total events, could otherwise lead to increased crosstalk.
