# Supplementary material for: 4D Super-Resolution Microscopy with Conventional Fluorophores and Single Wavelength Excitation in Optically Thick Cells and Tissues
Source: PLoS One. 2011 May 31;6(5):e20645. doi: 10.1371/journal.pone.0020645 (PMC3105105; doi:10.1371/journal.pone.0020645)
Supplement: Text S2 — Measurement of tissue autofluorescence. (PDF) [file pone.0020645.s006.pdf]

Measurement of tissue autofluorescence

Autofluorescence measurements were obtained by exciting a suspension of fixed isolated cardiac myocytes using a scanning monochrometer (Cornerstone 74000, Oriel, CT, USA) and a xenon arc lamp source (Oriel 66901, Oriel, CT, USA). Fluorescence emission was measured in a right-angle geometry using a USB spectrometer (USB2000+, Ocean Optics, FL, USA). Using a spectrometer for the detection allowed us to effectively reject signal due to scattered light from the fluorescence channel, and to use the amplitude of this scattered light peak to perform an in-situ correction for variations in the excitation intensity. To quantify autofluorescence, the signal was integrated over a 200 nm wide window starting 30 nm from the peak excitation wavelength. In this way we constructed an effective "excitation spectrum" of autofluorescence that quantifies the total spectral background intensity given excitation at a given wavelength between the near UV and the near infrared. The trend of the reduction in autofluorescence with increased excitation wavelength was captured by fitting a decaying exponential to the component of the signal above the noise floor of the spectrometer.
